# Dynamics of thrombin generation: Filling the gap between the system pharmacology theory and clinical practice in clinical pharmacology and therapeutics

**DOI:** 10.1002/prp2.70014

**Published:** 2024-12-31

**Authors:** Leire Ruiz, Sebastian Jaramillo, Andrea Calvo, M. A. Torrente, Dolors Tassies, J. C. Reverter, Annabel Blasi, Iñaki Troconiz

**Affiliations:** ^1^ University of Navarra Pamplona Spain; ^2^ Hospital Clinic Barcelona Barcelona Spain

**Keywords:** modeling, thombin generation

## Abstract

Mathematical models of thrombin generation (TG) that have been developed are based on a systems biology approach. Although this approach provides important information about the coagulation system, its clinical applicability is limited by its complexity and number of input variables required. The aim of this study was to develop a semimechanistic model able to describe TG in trauma and control patients. A dataset containing longitudinal data of TG assays and coagulation factors from 40 trauma patients and 20 control patients was used for model building. The model considered three fundamental processes: the degradation of tissue factor (TF) through a first‐order process, the activation of factor II by the TF through a first‐order process, and the degradation of thrombin through a first‐order process. Model fitting was performed using a nonlinear mixed‐effects approach. The condition of the patient (trauma and control) and coagulation factors were modelled as covariates. Model building demonstrated the presence of two additional processes that improved the predictive capacity of the model: the activation of factor II by TF governed by a second‐order constant and, a mechanism of factor II activation by TF characterized by a 7‐compartment transit chain governed by a second‐order constant. In the covariate model only the inclusion of patient condition was significant. Model evaluation demonstrated excellent performance in describing the temporal pattern of TG in trauma and control patients. Thrombin generation can be adequately modelled using a semimechanistic approach. Its application in practice could help to better assess the risk of hemorrhage and/or thrombosis in different settings.

## Graphical abstract

Mathematical model of thrombin generation build using a dataset containing TG assays and coagulation factors from 40 trauma patients and 20 control patients. The proposed semimechanistic model includes two processes: the activation of factor II by tissue factor governed by a second‐order constant, and factor II activation by a 7‐compartment transit chain governed by a second‐order constant.

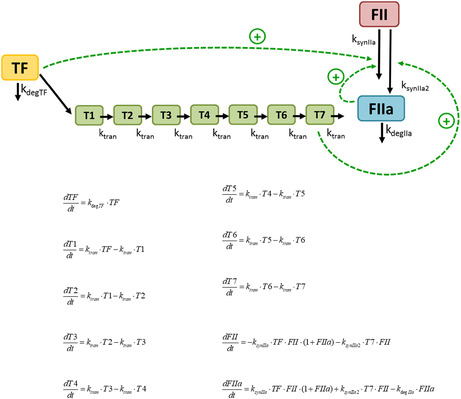

**Abbreviations**
AIC: akaike information criterion
AT: antithrombin
CATs: thrombin generation assays
CI: confidence intervals
CV: coefficient of variation
FII: factor II
FIIa: activated factor II
FIX: Factor IX
FVII: factor VII
FVIII: factor VIII
FX: Factor X
GOFs: goodness of fits plots
IIV: interindividual variability
OFV: objective function value
PPP: platelet‐poor plasma
RSE: relative standard errors
rTF: recombinant tissue factor
SCM: stepwise covariate modeling
TF: tissue factor
TG: thrombin generation
VI: factor V
VPCs: visual predictive checks

## 1 BACKGROUND

The coagulation system is an integrated biological mechanism that maintains the balance between pro and anticoagulant activities. Disorders in this balance lead to hemorrhagic or thrombotic complications, respectively.^1^, ^2^ The coagulation system is regulated by a strict homeostatic control that comprises two opposing drivers: the procoagulant drivers, triggered by tissue factor (TF) and the contact system, and the anticoagulant drivers triggered by activated protein C and antithrombin (AT), among others. The net result of the two pathways is the thrombin that ultimately converts fibrinogen to fibrin, making the blood clot.^3^, ^4^ The potential ability to synthesize thrombin is considered the best parameter to assess the global status of the complex coagulation system; it is made using the thrombin generation assay, which measures the concentration of thrombin produced over time after the addition of TF to a plasma sample. Thrombin generation is continuously monitored by means of a thrombin‐specific fluorogenic substrate, which shows the endogenous thrombin potential through a thrombin‐generation curve.^5^ However, this test is laborious and requires about an hour to be completed; additionally, the lack of standardization of the experimental conditions makes the comparison between curves generated from different experiments difficult, which limits its application in clinical practice.^6^

Given the complexity of thrombin generation and the limited applicability of tests to assess it, various mathematical models have been developed with the aim of understanding how this system is organized and controlled, how thrombin generation can be predicted and what analytical methods should be used for the diagnosis and treatment of patients.^7^ Most of these models are based on a systems biology approach, which aims to reproduce all the processes and factors that interact in thrombin generation. There are more than 80 published mathematical models, each with its own structure and number of parameters (https://doi.org/10.3390/pharmaceutics15030918). These models have been developed under a quantitative systems pharmacology or systems biology approach. These approaches are geared to develop purely mechanistic models, omitting the inter‐individual variability inherent in biological processes, and requiring a large number of parameters (usually over 30) to adequately describe the data. Although these models provide important information about the system, the huge number of components and processes involved limits their implementation in clinical practice.^8^ Semi‐mechanistic models could overcome this limitation as they reduce the number of system components while maintaining the key processes and factors of the system. Our semi‐mechanistic approach aimed to describe the data in a mechanistic way but at the same time incorporating the inter‐individual variability of the parameters, which allowed us to adequately describe thrombin formation with a smaller number of parameters.

However, to the best of our knowledge, no semi‐mechanistic model of thrombin generation has been developed.

The aim of this study was to develop a model to describe temporal thrombin generation in normal and trauma patients using a semi‐mechanistic approach. This would allow more personalized management of patients with a risk of bleeding and or thrombosis and aid clinical decision‐making in acute and chronic situations.

## 2 METHODS

### 2.1 Research data

Data from the study by Menezes et al.^9^ were used to develop the semi‐mechanistic model. The dataset provided by Menezes et al. included longitudinal data on thrombin generation assays (CATs) of 20 normal and 40 trauma patients, and the baseline levels of factors II, V, VII, VIII, IX, X, and antithrombin (AT) expressed in nM. The total number of thrombin observations (which refers to the number of thrombin measurements available for analysis; that is, the total count of thrombin measurements) was 1978 and 3736 for normal and trauma patients, respectively.

To analyze data a final concentration of 5 pM of recombinant tissue factor (rTFogether with 4 ÌM of procoagulant phospholipids was used. At this concentration of rTF, thrombin generation is dependent upon factors VIII and IX but not on factor XI, the influence of which is only seen at TF concentrations below 1 pM. In the 3 pM range and above, thrombin generation in PPP (platelet‐poor plasma) is not critically dependent on the rTF concentration, whereas around 1pM it is. rTF does not normally carry enough procoagulant phospholipids to ensure optimal prothrombin conversion. Thrombin generation is phospholipid dependent in the 0 to 2 ÌM range and reaches a plateau at F3 ÌM. At F10 ÌM, the contact activation properties of negatively charged PPL start to play a role, we therefore used 4 ÌM PPL. (Thrombinoscope™).^3^, ^5^

### 2.2 Data analysis

Longitudinal thrombin profiles were modeled using a nonlinear mixed‐effects approach. Mathematical fitting was performed in NONMEM (version 7.4; Icon Development Solutions, Hannover, MD, USA) using the First Order Conditional Estimation method with INTERACTION. Untransformed Thrombin data from normal and trauma patients were modeled simultaneously.

Interindividual variability (IIV) was modeled exponentially. The additive, proportional, and combined models were tested to describe residual variability.

### 2.3 Model building

An initial thrombin model was arranged on ordinary differential equations derived from a simplification of the models by Wajima et al. and Nayak et al.^10^, ^11^ This simplification was considered the base mechanistic framework to guide model building (Equation 1 and 2):

(1) $$\frac{\mathrm{dTF}}{\mathrm{dt}}=-k_{\text{degTF}}\times \mathrm{TF}$$

(2) $$\frac{\text{dFIIa}}{\mathrm{dt}}=k_{\text{synIIa}}\times \mathrm{TF}-k_{\text{degIIa}}\times \text{FIIa}$$

where the tissue factor (*TF*) (i) is degraded through a first‐order process represented by the first order rate constant *k*_degTF_ and (ii) activates factor II (*FIIa*) through a first‐order process (*k*_synIIa_). Thrombin degradation of thrombin is governed by a first‐order rate process (*k*_degIIa_).

### 2.4 Model selection

Comparison and discrimination of nested models were based on the objective function value (OFV) provided by NONMEM, approximately equal to a −2× log‐likelihood, where a decrease of 3.84 and 6.61 points in OFV between two nested model were associated to a 5 and 1% levels of significance, respectively.

Discrimination of non‐nested models was based on the Akaike information criterion (AIC), calculated as −2LL + 2 × NP, where NP is the number of parameters in the model.^12^ Additionally, parameter precision, assessed by relative standard errors (RSE) and the goodness of fits plots (GOFs)^13^ were also used as criteria for model selection and building. RSE were calculated as the ratio between the standard error and the estimate of the parameter.

### 2.5 Covariate selection

Covariate analysis was carried out once the base model was developed. The continuous covariates used in the analysis were *blood coagulation factors* (V, VII, VIII, IX, and X) and AT, expressed as percentages reported as supplementary material in the study by Menezes et al.^9^ exploring both linear and nonlinear relationships. The disease condition (normal or trauma) was analyzed as a categorical covariate.

Covariate selection made using the stepwise covariate modeling (SCM) implemented in the Perl‐speaks‐Nonmem (PsN) software (v.4.4.8),^14^ with a level of significance of 5% for the forward inclusion and 1% for the backward deletion, approaches.

### 2.6 Model evaluation

The thrombin model was evaluated through visual predictive checks (VPCs). A total of 500 datasets with the same characteristics as the original dataset were simulated. The 5th, 50th and 95th percentiles of simulated observations in each dataset were computed. Subsequently, the 90% confidence interval of each percentile calculated was obtained and plotted against the 5th, 50th, and 95th percentiles of raw thrombin data. Additionally, the precision of the parameter estimates was obtained from the analysis of 500 bootstrap datasets using PsN software.^14^

### 2.7 Statistical analyses

Comparison of the baseline levels of the different coagulation factor across normal and trauma patients were performed first exploring the normality of the distributions using the Shapiro–Wilk test. As the distributions of the afore mentioned factors differed statistically from the normal distribution, the nonparametric Wilcoxon rank test was used to evaluate statistical differences between groups of patients.

## 3 RESULTS

### 3.1 General description of the data

**FIGURE 1:**
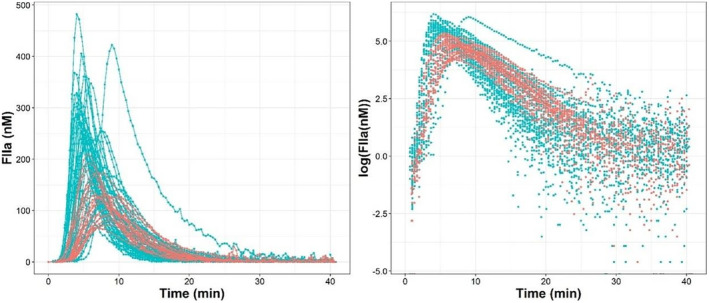
Individual profiles of thrombin generation observed. Blue and red lines and points represent normal and trauma subjects, respectively. Right panel shows logarithmic transformed thrombin of the data.

Figure 1 shows the individual thrombin profiles versus time with and without logarithmic transformation. A latency time was observed in both groups of patients, which was probably associated with all mechanisms preceding thrombin generation. In addition, a greater magnitude of noise was detected after 25 min. For this reason, it was decided to exclude data after 25 min from the analysis.

Table 1 provides a summary of the covariates in both groups of patients. Differences were observed between trauma and normal patients, reaching significance only for FVIII, probably due to the large variations in the data.

**TABLE 1:** Summary of coagulation factor values per subject condition.

Factors	Normal subjects		Trauma patients		Total individuals	
(nM)	Median	Min–Max	Median	Min–Max	Median	Min–Max
FII	1108.5	725.1–1338.06	1052.8	474.09–1784.83	1066.71	474.09–1784.83
FV	11.61	8.81–25.63	12.95	0.27–25.63	12.54	0.27–25.63
FVII	7.75	4.6–11.2	7.85	4.8–59.7	7.85	4.6–59.7
FVIII*	0.22	0.15–0.34	0.55	0.24–3.71	0.47	0.15–3.71
FIX	102.59	53.76–136.19	85.57	26.88–178.30	93.63	26.88–178.30
FX	129.85	78.44–162.1	129.85	61.01–207.42	129.85	61.01–207.42
AT	2805	2244–3332	2907	1768–5100	2873	1768–5100

* *p* < .0001 differences between normal and trauma patients based on the Wilcoxon test.

### 3.2 Mechanism‐based model

**FIGURE 2:**
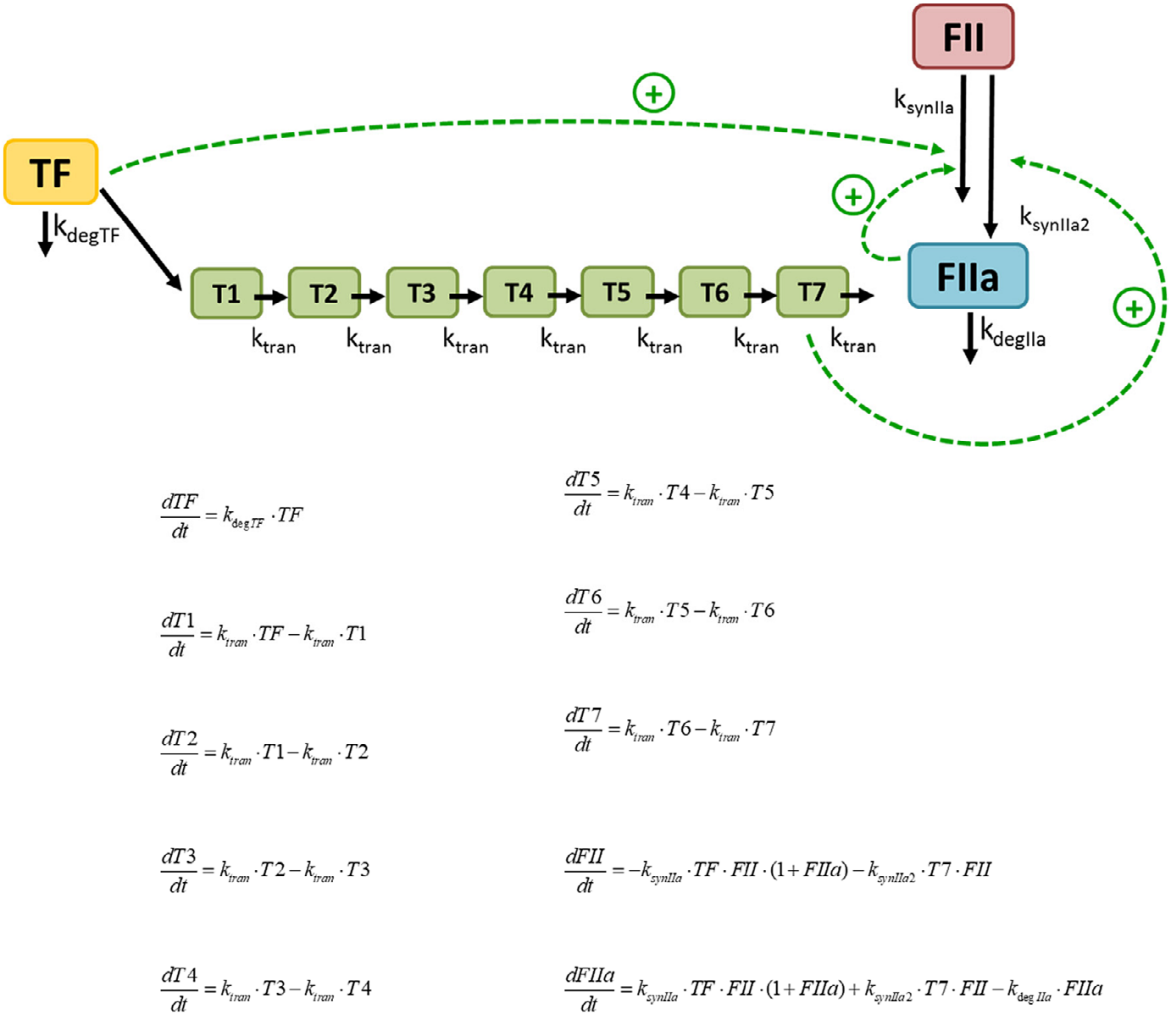
Schematic and mathematical representation of the model. Briefly, the model assumes that once TF is present: (i) a direct activation effect is triggered, characterized by the second order rate constant *ksynIIa* and, (ii) a second activation pathway is initiated which appears with a certain delay with respect to TF, characterized by a chain of seven transit compartments and the second order rate constant *ksynIIa2*. In addition, the model includes a regulatory mechanism depending on the thrombin generated, characterized by the parameter *kdegIIa*.

Figure 2 provides a schematic and mathematical representation of the model finally selected between different candidates, based on the previously described criteria.

The degradation process of TF represented by the first order rate constant *k*_degTF_, resulted negligible (*p* > .05) and was removed from the model, moreover, in an experimental period of 40 min, degradation of TF is unlike to occur, in fact the reported in vitro first‐order constant of the *in vitro* degradation rate is very small.^10^

The structure of the selected model comprised of seven transit compartments resembling and reducing the complexity of the coagulation cascade. In a first attempt a model allowing the estimation of both, the number of transit compartment and the mean transition time was fit to the data, those two parameters were identifiable. Then the number of transit compartments were added manually to the model until no further improvements based on the minimum value of the objective function were achieved. The selected model includes seven transit compartments.

Inter‐individual variability was found to be significant on all model parameters (*p* < .01). None of the off elements of the variance and covariance matrix resulted significant (*p* > .05). With respect the residual error, and given the fact that, as observed in Figure 1, experimental data, that values at times greater than 15 min show higher dispersion, a different estimate of residual variability was allowed for those observations. Up to 15 min after the start of the experiments residual variability was modeled with a combined error model, and beyond 15 min, the additive error model was selected.

After performing the forward inclusion and backward deletion approaches the only covariate included in the model was the individual condition (either normal or trauma patients) which affect to the *k*_synIIa_, *k*_degIIa_ and *k*_tran_ model parameters (*p* < .001). During the forward inclusion procedure factor VIII showed significant covariate effects on the parameter *k*_synIIa_ (*p* < .05), which vanished when subject condition was already present in the model. Nevertheless Figure S1 shows that no clear relationship between the parameter and factor VIII was observed. The current data did not support significant covariates effect of the coagulation factors measured at the baseline on any model parameter (*p* > .05).

Table 2 lists the estimates of model parameters and their corresponding precision, represented by 95% confidence interval computed from the bootstrap analysis. None of the estimates of the 95% confidence intervals included the zero value, indicating that parameters were significant for the model. All estimates were within the 95% confidence interval obtained by bootstrapping, demonstrating the robustness of the model.

**TABLE 2:** Estimates of the model parameters corresponding to the selected model.

Parameter (units)	Estimate (95% CI)	IIV (95% CI)	Shrinkage (%)
ksynIIa (nM^−1^ × min^−1^)	Normal = 4.7 × 10^−3^ (2 × 10^−4^–1.8 × 10^−2^) Trauma = 0.15 (0.12–0.18)	240 (145–630) 51 (30–90)	63 25
kdegIIa (min^−1^)	Normal = 1.04 (0.95–1.14) Trauma = 0.8 (0.74–0.86)	20 (14–25) 25 (20–28)	38 14
ktran (min^−1^)	Normal = 1.4 (1.24–1.53) Trauma = 1.8 (1.6–2.1)	23 (17–26) 42 (11–48)	38 14
ksynII2a (nM^−1^ × min^−1^)	Normal = 47 (43.5–50.2) Trauma = 45 (39.9–49.9)	14 (4.5–17.3) 34 (25–39)	41 18
Additive error (nM)	Time < 15 min = 2.4 (1.3–2.8) Time > 15 min = 2.4 (2–3.1)	— —	2 2
Proportinal error (%)	0.18 (0.14–0.21)	—	2

*Note*: Estimates of interindividual variability (IIV) are expressed in coefficient of variation (CV(%)), calculated $\mathrm{CV}\left(\%\right)=\sqrt{\omega^{2}}\times 100$, where *ω* ^2^ represents the variance of the corresponding random effects. Parameter precision is represented by the 95% confidence intervals (95% CI) obtained from the analysis of 500 hundred bootstrap datasets.

**FIGURE 3:**
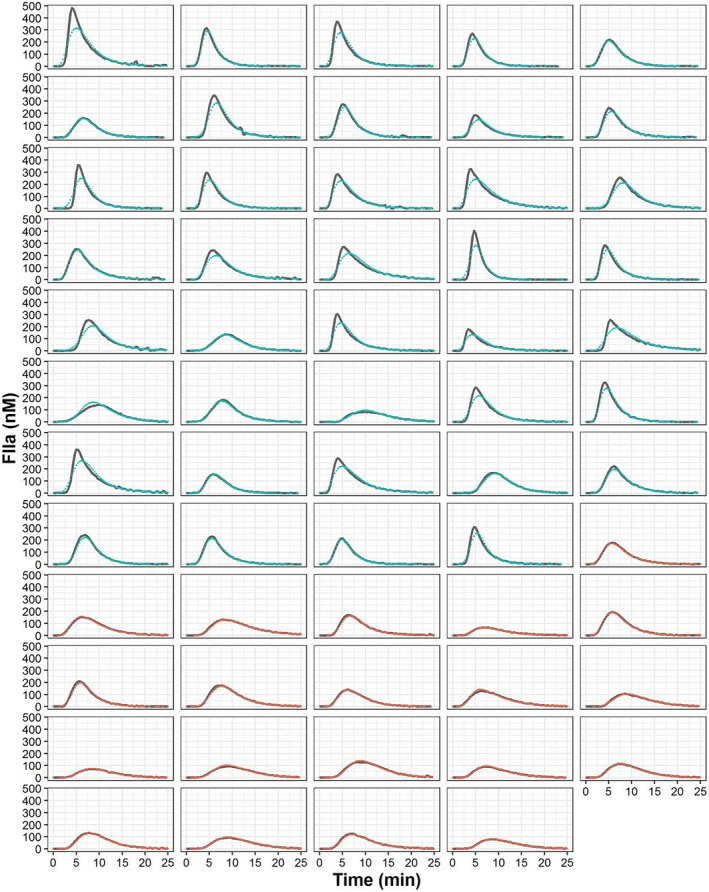
Individual thrombin observations (red dots, normal subjects; blue dots, trauma patients) and individual model predictions (gray lines) over time.

Figure 3 shows the individual observed and model predicted profiles indicating an excellent model performance at the individual level. Figure S2 shows the goodness of fit plots (upper panels) and the results of the VPCs (lower panels) corresponding to thrombin profile stratify by the group of patients (normal and trauma). The model performed adequately in capturing the central trend and the dispersion of the data.

## 4 DISCUSSION

In this study, we developed a semi‐mechanistic model that described the time course of thrombin generation in normal and trauma patients. Thrombin generation was characterized by a mechanism of direct activation of TF on FII, an indirect mechanism of TF on FII that included 7 consecutive processes, a positive feedback mechanism of thrombin on FII and a mechanism of thrombin degradation. All these mechanisms were estimated separately according to the patient condition (normal or trauma). The inclusion of coagulation factors as parameter covariates was not significant.

Semi‐mechanistic models are useful alternatives to systems biology‐based models, which allow the key processes and mechanisms of the system to be maintained without affecting the predictive capacity. In this line, the model developed in this study has considerably reduced complexity and number of parameters compared with other published models. Although not properly compared in this study, the model developed demonstrated a predictive capability equivalent to systems biology models, as shown in the performance plots (Figure 3 and Figure S2).

The semimechanistic model developed describes two different mechanisms for thrombin generation. The reaction ruled by the *k*_synIIa_ parameter (responsible for a quick burst of thrombin) corresponds to the FXa reaction in the models by Wajima et al. and Nayak et al.^10^, ^11^ On the other hand, the *k*_synIIa2_ parameter, responsible for generating large thrombin concentrations corresponds to the Xa:Va reaction in the models by Wajima et al. and Nayak et al.^10^, ^11^ These two mechanisms are in agreement with the cell‐based coagulation model proposed by Hoffmann et al.^3^ in which the first thrombin synthesis corresponds to the initiation phase and the second to the propagation phase. Nevertheless, because of differences in the structure of the models, it is difficult to compare parameter estimates, even though the mechanisms and entities involved are similar. The thrombin degradation rate constant (*kdegIIa*) was the only comparable parameter that provided similar values for the developed model and the model by Wajima et al. (1.04 min^−1^ for normal subjects, and 1.12 min^−1^, respectively).^10^ Gulati et al. obtained a comparable value for the degradation rate constant of thrombin (0.97 min^−1^).^15^ These authors reduced the systems biology model by Wajima et al.^10^ through clustering in order to estimate the parameters to describe the fibrinogen concentrations versus time profiles obtained from the data of patients with venom‐induced consumption coagulopathy.

With respect to the *kdegTF* parameter, it was fixed to 0 in the model developed because of the assumed slow degradation rate “in vitro” described in the model by Wajima et al. (reflected by a half‐life of 831 min) compared to the length of the experimental procedure (40 min).^10^

The model developed has some limitations. Firstly, the analysis was performed in a small fraction of the population, including only normal subjects and trauma patients, and with a high between‐subject variability. This limitation compromises model predictability and generalization. Nevertheless, the simulations performed with the VPCs suggest that the model performs adequately and predicts the raw data well. The second limitation is that the model was built based on data from Menezes et al.^9^ which only provided the concentration for some coagulation factors. Including the concentration of activated factors may represent an opportunity to discern between normal subjects and traumatized patients and thus provide more accurate predictions based on the patient's condition.

The model developed by our group requires the values of FII and TF to predict FIIa. During model building (both in structural model development and covariate analysis), we tested whether the inclusion of other coagulation factors resulted in a better model fit. However, none of the coagulation factors demonstrated a significant improvement, indicating that FIIa formation can be adequately described with information regarding FII and TF alone. The effect of fibrinogen was showed by adding increasing concentrations of fibrinogen to afibrinoginemic plasma, where fibrinogen dose‐dependently increases the amount of thrombin in a fluorometer experiment. It could entails an inherent study limitation, since the variability coming from the fibrinogen plasma levels cannot be considered. To the contrary, similar TF levels were used to generate TG in all subjects. However, although we cannot verify it, it is likely that the activation and action of factors not included in the model are implicitly represented in the model's transit compartments.

From a clinical point of view, these results are relevant for several reasons. Firstly, simplicity, while maintaining the effectivity of a model describing temporal thrombin generation: namely, if we replicated our model with data from Menezes et al., we would come to similar and easier conclusions. Second, this opens the opportunity to develop point‐of‐care devices to measure a few coagulation factors and estimate TG. This would allow patients to be stratified according to the risk of bleeding/thrombosis at some point in both standard and emergent clinical settings. Finally, this model (although constructed using on healthy subjects and trauma patients) could be extrapolated to different patient populations but not without first testing it in future studies. It will be particularly interesting to check how the model performs in patients with quantitative and qualitative changes in several coagulation factors, such as patients with liver disease.

In conclusion, systems biology models are very useful to understand the coagulation system: however, their complexity, number of parameters and number of input variables limit their clinical applicability. The strength of the present work is to provide a simplified approach that facilitates clinical applicability. The semi‐mechanistic model developed may provide important information for personalized patient treatment.

## AUTHOR CONTRIBUTIONS

A.B., L.R., and I.T. wrote the manuscript; A.B., D.T., JC.R., and I.T designed the research; A. C., MA. T., and S.J performed the research; S.J. and I.T. analyzed the data.

## ETHICS STATEMENT

The study was conducted in accordance with the Declaration of Helsinki.

## DISCLOSURE

All authors have no conflict of interest to disclose.

## Supporting information


Figure S1.

## Data Availability

The data that support the findings of this study are available from the corresponding author upon reasonable request.
