# Supplementary material for: Dynamics of thrombin generation: Filling the gap between the system pharmacology theory and clinical practice in clinical pharmacology and therapeutics
Source: Pharmacol Res Perspect. 2024 Dec 31;13(1):e70014. doi: 10.1002/prp2.70014 (PMC11687557; doi:10.1002/prp2.70014)
Supplement: Supplementary file 1 — Figure S1. [file PRP2-13-e70014-s001.docx]

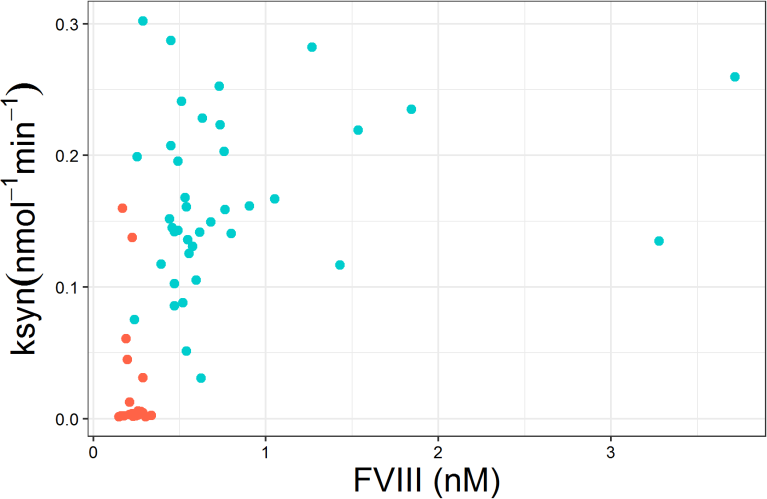


**Figure 1S.** Graphic representation of the relationship between the *ksynIIa* parameter and FVIII for normal subjects (red) and trauma patients (blue). After visual exploration, it was concluded that no significant relationship could be established.


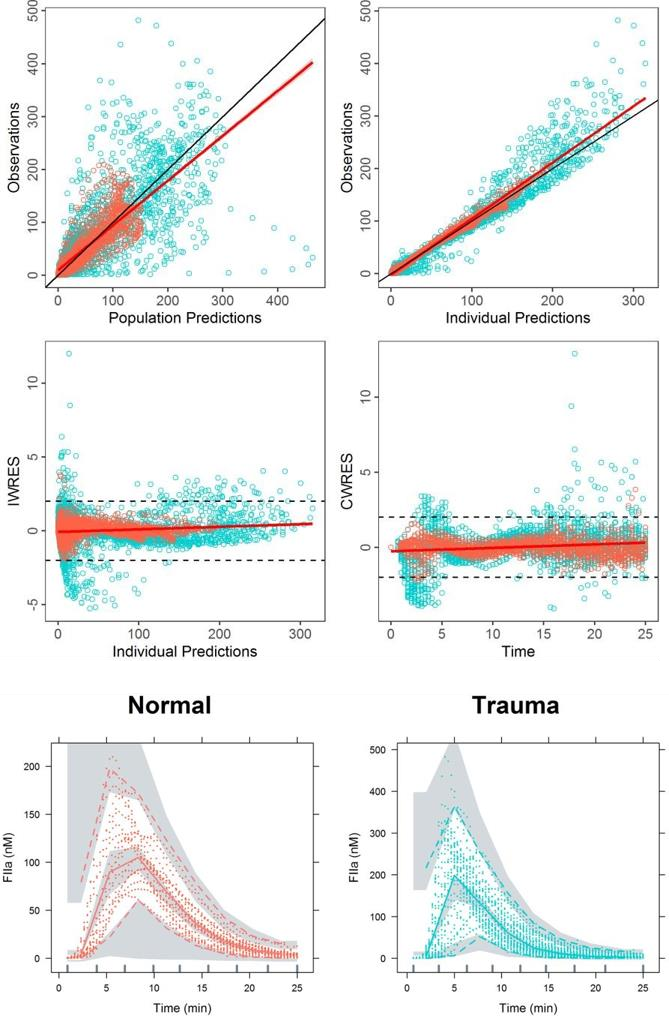


**Figure 2S**. (Up) Goodness of fit plots corresponding to the semi-mechanistic model selected. Circles are the observed data (red normal and blue trauma). Black lines represent the perfect fit. Solid red lines represent a smooth curve through the data. (Down) VPCs of thrombin profiles in normal and trauma patients. Red and blue dots represent thrombin observations; the solid red and blue lines correspond to the median of the observed data while the dashed red and blue lines the 5 and 95

percentiles of the observations. Shaded grey areas are the 90% predicted intervals for corresponding percentiles obtained from 500 simulated studies.
